# Dissection of neuroinflammation in schizophrenia

**DOI:** 10.1192/bjo.2021.730

**Published:** 2021-06-18

**Authors:** Fizah Muratib, Yuya Mizuno, Ines Carreira Figueiredo, Oliver Howes, Tiago Reis Marques

**Affiliations:** IoPPN King's College London

## Abstract

**Aims:**

Schizophrenia is notoriously becoming one of the world's most debilitating mental disorders, affecting 1 in 100 people. There is increasing evidence that neuroinflammation plays a part in the pathogenesis of schizophrenia and other psychotic disorders; microglial activity acting as a marker for neuroinflammatory reactions in the brain. Furthermore, cannabis is an illicit substance that also evokes a similar response in the neuroimmune activity. This project explores how cannabis exposure influences an elevation in neuroinflammatory responses through TSPO levels, and whether this information can help us determine if cannabis use and increased TSPO levels can be associated with a risk factor for developing psychosis.

**Method:**

55 participants (36 males and 19 females) were recruited from the community by the IRIS (Inflammatory Reaction in Schizophrenia) team at the IoPPN, King's College London, from which 34 patients with a diagnosis of schizophrenia and 21 healthy controls took part in the study. The eligible participants underwent clinical assessments and PET scanning, from which cannabis use history and PET data were collected. Participant neuroinflammatory levels are represented by [18F]DPA-714 volume and different regions of grey matter in the brain were analysed through multivariate analyses, the confounding variables being age and TSPO genotype.

**Result:**

A statistically significant association is shown between participants who have had exposure to cannabis and participants who have not had any exposure in their lifetime. The differences across the prioritised brain regions of interest were robust, the association appearing more apparent and statistically significant in the total (p = .00) and temporal grey matter (p = .00) regions of the brain. This may suggest that cannabis exposure influences the [18F]DPA-714 VT in the significant regions of interest. However, a negative association is seen with current use, the quantity of use, and the frequency of use.

**Conclusion:**

The initial findings for cannabis exposure show us a positive association with increased TSPO levels, however, limitations must be taken into account. Although we cannot readily establish that elevated TSPO levels in cannabis users can presently act as a risk factor marker for developing psychosis from this particular study, we can utilise this data to continue our research in disclosing a new system to predict the occurrence of psychosis.

